# Supplementary material for: COZOID: contact zone identifier for visual analysis of protein-protein interactions
Source: BMC Bioinformatics. 2018 Apr 6;19:125. doi: 10.1186/s12859-018-2113-6 (PMC5889581; doi:10.1186/s12859-018-2113-6)
Supplement: Supplementary file 2 — Software build. Executable binary file of the software tool. (ZIP 118,784 kb) [file 12859_2018_2113_MOESM2_ESM.zip › cozoid/platform/docs/testng-6.5.1-javadoc.zip!/org/testng/TestNG.ExitCodeListener.html]

TestNG.ExitCodeListener (TestNG)


---


|  |  |  |
| --- | --- | --- |
| **Overview** \| **Package** \| **Class** \| **Tree** \| **Deprecated** \| **Index** \| **Help** | |  |
| **PREV CLASS**   **NEXT CLASS** | **FRAMES**    **NO FRAMES**     **All Classes** |  |
| SUMMARY: NESTED \| FIELD \| CONSTR \| METHOD | DETAIL: FIELD \| CONSTR \| METHOD |  |


---


## org.testng Class TestNG.ExitCodeListener

```
java.lang.Object
  org.testng.TestNG.ExitCodeListener
```

**All Implemented Interfaces:**: IConfigurationListener, IConfigurationListener2, org.testng.internal.IResultListener, org.testng.internal.IResultListener2, ITestListener, ITestNGListener

**Enclosing class:**: TestNG

---

``` public static class TestNG.ExitCodeListener extends java.lang.Object implements org.testng.internal.IResultListener2 ```

---

**Constructor Summary**

`TestNG.ExitCodeListener()`

`TestNG.ExitCodeListener(TestNG runner)`


| **Method Summary** | |
| --- | --- |
| `void` | `beforeConfiguration(ITestResult tr)`             Invoked before a configuration method is invoked. |
| `void` | `onConfigurationFailure(ITestResult itr)`             Invoked whenever a configuration method failed. |
| `void` | `onConfigurationSkip(ITestResult itr)`             Invoked whenever a configuration method was skipped. |
| `void` | `onConfigurationSuccess(ITestResult itr)`             Invoked whenever a configuration method succeeded. |
| `void` | `onFinish(ITestContext context)`             Invoked after all the tests have run and all their Configuration methods have been called. |
| `void` | `onStart(ITestContext context)`             Invoked after the test class is instantiated and before any configuration method is called. |
| `void` | `onTestFailedButWithinSuccessPercentage(ITestResult result)`             Invoked each time a method fails but has been annotated with successPercentage and this failure still keeps it within the success percentage requested. |
| `void` | `onTestFailure(ITestResult result)`             Invoked each time a test fails. |
| `void` | `onTestSkipped(ITestResult result)`             Invoked each time a test is skipped. |
| `void` | `onTestStart(ITestResult result)`             Invoked each time before a test will be invoked. |
| `void` | `onTestSuccess(ITestResult result)`             Invoked each time a test succeeds. |

**Methods inherited from class java.lang.Object**

`clone, equals, finalize, getClass, hashCode, notify, notifyAll, toString, wait, wait, wait`

**Constructor Detail**

### TestNG.ExitCodeListener

```
public TestNG.ExitCodeListener()
```

---


### TestNG.ExitCodeListener

```
public TestNG.ExitCodeListener(TestNG runner)
```


**Method Detail**

### beforeConfiguration

```
public void beforeConfiguration(ITestResult tr)
```

:   **Description copied from interface: `IConfigurationListener2`**
:   Invoked before a configuration method is invoked.

    :   **Specified by:**: `beforeConfiguration` in interface `IConfigurationListener2`

---


### onTestFailure

```
public void onTestFailure(ITestResult result)
```

:   **Description copied from interface: `ITestListener`**
:   Invoked each time a test fails.

    :   **Specified by:**: `onTestFailure` in interface `ITestListener`
    :   **Parameters:**: `result` - `ITestResult` containing information about the run test **See Also:**: `ITestResult.FAILURE`

---


### onTestSkipped

```
public void onTestSkipped(ITestResult result)
```

:   **Description copied from interface: `ITestListener`**
:   Invoked each time a test is skipped.

    :   **Specified by:**: `onTestSkipped` in interface `ITestListener`
    :   **Parameters:**: `result` - `ITestResult` containing information about the run test **See Also:**: `ITestResult.SKIP`

---


### onTestFailedButWithinSuccessPercentage

```
public void onTestFailedButWithinSuccessPercentage(ITestResult result)
```

:   **Description copied from interface: `ITestListener`**
:   Invoked each time a method fails but has been annotated with
    successPercentage and this failure still keeps it within the
    success percentage requested.

    :   **Specified by:**: `onTestFailedButWithinSuccessPercentage` in interface `ITestListener`
    :   **Parameters:**: `result` - `ITestResult` containing information about the run test **See Also:**: `ITestResult.SUCCESS_PERCENTAGE_FAILURE`

---


### onTestSuccess

```
public void onTestSuccess(ITestResult result)
```

:   **Description copied from interface: `ITestListener`**
:   Invoked each time a test succeeds.

    :   **Specified by:**: `onTestSuccess` in interface `ITestListener`
    :   **Parameters:**: `result` - `ITestResult` containing information about the run test **See Also:**: `ITestResult.SUCCESS`

---


### onStart

```
public void onStart(ITestContext context)
```

:   **Description copied from interface: `ITestListener`**
:   Invoked after the test class is instantiated and before
    any configuration method is called.

    :   **Specified by:**: `onStart` in interface `ITestListener`

---


### onFinish

```
public void onFinish(ITestContext context)
```

:   **Description copied from interface: `ITestListener`**
:   Invoked after all the tests have run and all their
    Configuration methods have been called.

    :   **Specified by:**: `onFinish` in interface `ITestListener`

---


### onTestStart

```
public void onTestStart(ITestResult result)
```

:   **Description copied from interface: `ITestListener`**
:   Invoked each time before a test will be invoked.
    The `ITestResult` is only partially filled with the references to
    class, method, start millis and status.

    :   **Specified by:**: `onTestStart` in interface `ITestListener`
    :   **Parameters:**: `result` - the partially filled `ITestResult` **See Also:**: `ITestResult.STARTED`

---


### onConfigurationFailure

```
public void onConfigurationFailure(ITestResult itr)
```

:   **Description copied from interface: `IConfigurationListener`**
:   Invoked whenever a configuration method failed.

    :   **Specified by:**: `onConfigurationFailure` in interface `IConfigurationListener`
    :   **See Also:**: `IConfigurationListener.onConfigurationFailure(org.testng.ITestResult)`

---


### onConfigurationSkip

```
public void onConfigurationSkip(ITestResult itr)
```

:   **Description copied from interface: `IConfigurationListener`**
:   Invoked whenever a configuration method was skipped.

    :   **Specified by:**: `onConfigurationSkip` in interface `IConfigurationListener`
    :   **See Also:**: `IConfigurationListener.onConfigurationSkip(org.testng.ITestResult)`

---


### onConfigurationSuccess

```
public void onConfigurationSuccess(ITestResult itr)
```

:   **Description copied from interface: `IConfigurationListener`**
:   Invoked whenever a configuration method succeeded.

    :   **Specified by:**: `onConfigurationSuccess` in interface `IConfigurationListener`
    :   **See Also:**: `IConfigurationListener.onConfigurationSuccess(org.testng.ITestResult)`


---


|  |  |  |
| --- | --- | --- |
| **Overview** \| **Package** \| **Class** \| **Tree** \| **Deprecated** \| **Index** \| **Help** | |  |
| **PREV CLASS**   **NEXT CLASS** | **FRAMES**    **NO FRAMES**     **All Classes** |  |
| SUMMARY: NESTED \| FIELD \| CONSTR \| METHOD | DETAIL: FIELD \| CONSTR \| METHOD |  |


---
